# Immunization with mutant HPV16 E7 protein inhibits the growth of TC-1 cells in tumor-bearing mice

**DOI:** 10.3892/ol.2015.2911

**Published:** 2015-01-28

**Authors:** YAN-LI LI, ZHONG-LIANG MA, YUE ZHAO, JING ZHANG

**Affiliations:** 1 School of Life Sciences, Shanghai University, Shanghai 200444, P.R. China; 2 Institute of Molecular Medicine, Nanjing University, Nanjing, Jiangsu 210093, P.R. China

**Keywords:** human papillomavirus, mutant E7 protein, immunization, TC-1 cells

## Abstract

Two human papillomavirus (HPV) 16 oncogenic proteins, E6 and E7, are co-expressed in the majority of HPV16-induced cervical cancer cells. Thus, the E6 and E7 proteins are good targets for developing therapeutic vaccines for cervical cancer. In the present study, immunization with the mutant non-transforming HPV16 E7 (mE7) protein was demonstrated to inhibit the growth of TC-1 cells in the TC-1 mouse model. The HPV16 mE7 gene was amplified by splicing overlap extension polymerase chain reaction using pET-28a(+)-E7 as a template, and the gene was cloned into pET-28a(+) to form pET-28a(+)-mE7. Compared with the E7 protein, mE7 lacks amino acid residues 94–98, and at residue 24, there is a Cys to Gly substitution. pET-28a(+)-mE7 was then introduced into *Escherichia coli* Rosetta. The expression of mE7 was induced by isopropyl β-D-1-thiogalactopyranoside. The mE7 protein was purified using Ni-NTA agarose and detected by SDS-PAGE and western blot analysis. In the tumor prevention model, no tumor was detected in the mice vaccinated with the mE7 protein. After 40 days, the tumor-free mice and control mice were challenged with 2×10^5^ TC-1 cells. All control mice developed tumors six days later, but mE7 immunized mice were tumor free until 90 days. In the tumor therapy model, the TC-1 cells were initially injected subcutaneously, and the mice were subsequently vaccinated. Vaccination against the mE7 protein may significantly inhibit TC-1 cell growth compared to the control. These results demonstrated that immunization with the HPV16 mE7 protein elicited a long-term protective immunity against TC-1 tumor growth and generated a significant inhibition of TC-1 growth in a TC-1 mouse model.

## Introduction

Worldwide, cervical cancer is the third most frequently diagnosed cancer and is the fourth leading cause of cancer-associated mortality in females, accounting for 9% of all novel cancer cases and 8% of all cancer-associated mortalities in females in 2008 (1). The development of cervical cancer is associated with recurrent high-risk human papillomavirus (HPV) infection, particularly HPV16, which exhibits a prevalence of ~60% (2–5). The E6 and E7 proteins of HPV16 play an important role in the occurrence and development of HPV16-induced cervical cancer (6). Therefore, the E6 and E7 oncogenic proteins are good targets for developing therapeutic vaccines for cervical cancer (7,8). Immunization with E6 or E7 long peptide-containing human leukocyte antigen epitopes or recombinant proteins may generate potent antitumor immunity (9–14). In previous studies, it has been found that immunization with the HPV16 E7 protein elicited specific protective immunity against TC-1 tumor growth (15), and mouse autologous heat shock protein 70 (HSP70) enhanced the antitumor potency of the E7 protein without any adjuvant (16).

It has been reported that mutations in the E6 or E7 proteins severely reduces or completely abolishes the transforming ability of these proteins (17–19). His-2, Cys-24 and two Cys-X-X-Cys metal-binding motifs, located at amino acid residues 58–61 and 91–94, are the most important regions associated with the transforming ability of the E7 protein (19). Focal transformation of rat 3Y1 cells by E7 has been demonstrated to be eliminated by His-2 to Asp or Cys-24 to Gly mutations and the transformation was also considerably impaired by Cys-61 or Cys-94 to Gly mutations (19). In order to improve the safety of the protein vaccine, the mutation in the E7 sequence was cloned in this study. Compared with the E7 gene, mutant non-transforming HPV16 E7 (mE7) contains a T to G point mutation at base 70 and removal of 15 bases from the 3′ end of the E7 protein, which substitutes Cys-24 with Gly and disrupts the carboxyl terminal Cys-X-X-Cys motif by deleting five amino acid residues that contain the code for Cys-94 in the E7 protein.

Protein vaccination has become the most popular form of HPV therapeutic vaccines as the vaccines are safe and demonstrate no human leukocyte antigen restriction (20). In the present study, the mutant HPV16 E7 gene (mE7) was amplified by splicing overlap extension polymerase chain reaction (splicing PCR) using pET-28a(+)-E7 as a template. The mE7 protein was expressed and purified in an *Escherichia coli (E. coli)* system. The inhibition of TC-1 cell growth was then investigated using a TC-1 mouse model (21). TC-1 cells derived from primary epithelial cells of C57BL/6 mice were co-transformed with the HPV16 E6, E7 and c-Ha-ras oncogenes. The TC-1 mouse model is a popular model of human cervical cancer. In the present study, mice were immunized with the mE7 protein to investigate the inhibition of TC-1 cell growth by using this model.

## Materials and methods

### Materials

*E. coli* DH5α and Rosetta were purchased from Invitrogen (Carlsbad, CA, USA). Yeast extract and tryptone were obtained from Thermo Fisher Scientific (Waltham, MA, USA). RPMI-1640 medium, fetal bovine serum, non-essential amino acids and G418 were obtained from Gibco Life Technologies (Grand Island, NY, USA). Plas/mini Isolation Spin-kit, complete Freund’s adjuvant (CFA) and incomplete Freund’s adjuvant (IFA) were obtained from Sigma-Aldrich (St. Louis, MO, USA). Sodium bicarbonate and sodium pyruvate were obtained from Amresco LLC (Solon, OH, USA). Taq DNA polymerase, T4 DNA ligase, restriction endonucleases and pMD18T vector were obtained from Takara Biotechnology Co., Ltd. (Dalian, Liaoning, China). L-glutamine was purchased from HyClone (Logan, UT, USA). N-2-Hydroxyethylpiperazine-N′-2-ethanesulfonic acid (HEPES) was obtained from Promega (Madison, WI, USA). Penicillin was purchased from North China Pharmaceutical Group (Shijiazhuang, Hebei, China), streptomycin was from Shandong Lukang Pharmaceutical Co. (Jining, Shandong, China) and the expression vector pET-28a(+) was from Novagen (Darmstadt, Germany). Ni-NTA agarose was obtained from Qiagen (Valencia, CA, USA). The HPV16 E7 antibody, a mouse monoclonal IgG1, was purchased from Santa Cruz Biotechnology (cat. no. sc-6981; Dallas, TX, USA) and the PCR primers were synthesized by Shanghai Sangon Biological Engineering Technology and Service Co., Ltd. (Shanghai, China).

### Cell culture

The TC-1 cell line was provided by Dr Wu of Johns Hopkins University (Baltimore, MD, USA). The cells were maintained in RPMI-1640, supplemented with 10% fetal bovine serum, 400 μg/ml G418, 2 mM L-glutamine, 1.5 mg/ml sodium bicarbonate, 4.5 mg/ml glucose, 10 mM HEPES, 1 mM sodium pyruvate, 2 mM non-essential amino acid, 100 units/ml penicillin and 100 μg/ml streptomycin at 37°C in a 5% CO_2_ atmosphere.

### Construction of the pET-28a(+)-mE7 expression plasmid

In a previous study, the HPV16 E7 gene was cloned from the human cervical cancer cell line CaSKi and the prokaryotic expression vector pET-28a(+)-E7 was constructed (15). The HPV16 mE7 gene was amplified by splicing PCR using pET-28a(+)-E7 as a template. Two sets of primers were used. The first set of primers was the forward primer mE7p1 that contained a site for the restriction enzyme *Nde*I and the reverse primer mE7p1-G-3′, which carried a T to G mutation at base 70 in the E7 gene (Table I). The primers were used to amplify the sequence between the *Nde*I site and base 84 (135 bp; *Nde*I-E7_84_). The second set of primers was the forward primer mE7p2-G-5′, which carried a T to G mutation at base 70 in the E7 gene, and the reverse primer mE7p2, which contained a site for *Hind*III (Table I). The second set of primers was used to amplify the E7 gene between bases 56 and 279, with a UAA stop codon (236 bp; E7_56–279_). The two PCR products *Nde*I-E7_84_ and E7_56–279_ were purified by electrophoresis in 2% agarose gel. mE7 was then amplified by splicing PCR using *Nde*I-E7_84_ and E7_56–279_ as templates. The primers were mE7p1 and mE7p2.

PCR was then performed as previously described (22). The conditions for PCR amplification were as follows: 94°C for 3 min; 35 cycles of 94°C for 45 sec, 56°C for 1 min, and 72°C for 45 sec; and a final extension for 3 min at 72°C. The recombinant pMD18T-mE7 and pET-28a(+)-mE7 plasmids were constructed as previously described (15).

### Expression, purification and characterization of the mE7 protein

Expression of the mE7 protein was assessed as previously described (15,16). Briefly, the expression of mE7 was induced in *E. coli* Rosetta by isopropyl-β-D-thiogalactoside (IPTG). The inclusion body and supernatant were analyzed by SDS-PAGE in a 12% agarose gel. The inclusion bodies that contained the mE7 protein were then dissolved, and the mE7 protein was purified using Ni-NTA agarose column chromatography. The identity and the purity of the recombinant proteins were determined by SDS-PAGE. The concentration of the proteins was measured using a Bradford assay (23). To confirm the characteristics of the mE7 protein, the human HPV16 E7 antibody was used to verify the purified protein using western blot analysis.

### Prevention and inhibition of TC-1 cell growth by immunization with the mE7 protein

The tumor model was established using female C57BL/6 mice that were 6–8 weeks old, obtained from Shanghai Laboratory Animal Center (Shanghai, China). All procedures performed in the animal study were approved by the Animal Study Committee of the Institute of Molecular Medicine (Nanjing University, Nanjing, China).

The preventative tumor model was established following the previously described method (15,16). In brief, 12 mice underwent subcutaneous (SC) immunization by either PBS or 1.5 nmol mE7 protein with CFA. A second equivalent dose of the same solution with IFA was administered by intraperitoneal injection two weeks later. The mice received an additional SC injection, consisting of 1×10^5^ TC-1 cells, in the right flank seven days subsequent to the second immunization. After 40 days, the tumor-free mice and a novel group of control mice were again challenged with 2×10^5^ TC-1 cells. The tumor growth was monitored and tumor incidence was also recorded.

In the therapeutic tumor model, 30 mice were administered with an SC injection consisting of 1×10^5^ TC-1 cells in the right flank, and underwent SC immunization with 1.5 nmol mE7 protein, E7 protein or PBS in combination with CFA on the following day. Each group contained 10 mice. One week later, the mice were immunized by intraperitoneal injection using the same dose of protein supplemented with IFA. Tumor growth was monitored every three days until the control mice began to succumb. The survival rate was recorded and the tumor volume was determined as previously described (15).

### Statistical analysis

The statistical analysis was performed as previously described (15). All data were expressed as the mean ± standard error of the mean. The comparison of tumor volume between individual data points was made using a Student’s t-test. The data for the survival percentage were evaluated by the Log-rank test. P<0.05 was considered to indicate a statistically significant difference.

## Results

### Construction of pET-28a(+)-mE7 plasmid

**Figure 1:**
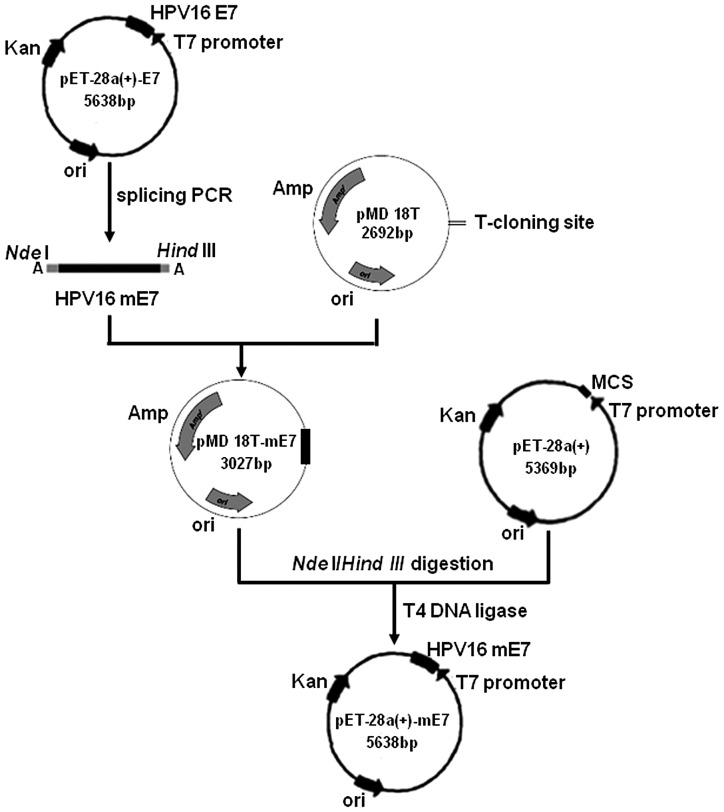
Construction of the pET-28a(+)-mE7 plasmid. The HPV16 mE7 gene was amplified by splicing overlap extension PCR using pET-28a(+)-E7 as a template. The mE7 gene was then directly inserted into a pMD18T vector to form pMD18T-mE7. The pET-28a(+) and pMD18T-mE7 vectors were each digested with restriction endonucleases *Nde*I and *Hind*III. Subsequent to purification with electrophoresis in a 2% agarose gel, mE7 and pET-28a(+) were ligated with T4 DNA ligase to yield the expression plasmid pET-28a(+)-mE7. PCR, polymerase chain reaction; HPV, human papillomavirus; ori, origin of replication; mE7, mutant non-transforming HPV16 E7.

The procedure for the construction of the pET-28a(+)-mE7 plasmid is shown in Fig. 1. The HPV16 mE7 gene was amplified by splicing PCR using pET-28a(+)-E7 as a template. The mE7 gene was then directly inserted into a pMD18T vector to form the pMD18T-mE7 plasmid. The pET-28a(+) and pMD18T-mE7 vectors were each digested with the *Nde*I and *Hind*III restriction endonucleases. Subsequent to purification by electrophoresis on a 2% agarose gel, mE7 and pET-28a(+) were ligated with T4 DNA ligase to yield the pET-28a(+)-mE7 expression plasmid. Compared with the E7 gene, mE7 was lacking nucleotides 280–294, which corresponds to residues 94–98 in the E7 protein, and nucleotide 70 in mE7 is changed from T to G, which results in residue 24 in the E7 protein being changed from Cys to Gly. The construction of the pET-28a(+)-mE7 plasmid allowed the expression of the fusion mE7 protein to have a hexa-histidine tag (his-tag) at the N-terminal end. The his-tag sequence served as a tag for affinity purification using a Ni-NTA agarose column.

### Expression, purification and characterization of the mE7 protein

**Figure 2:**
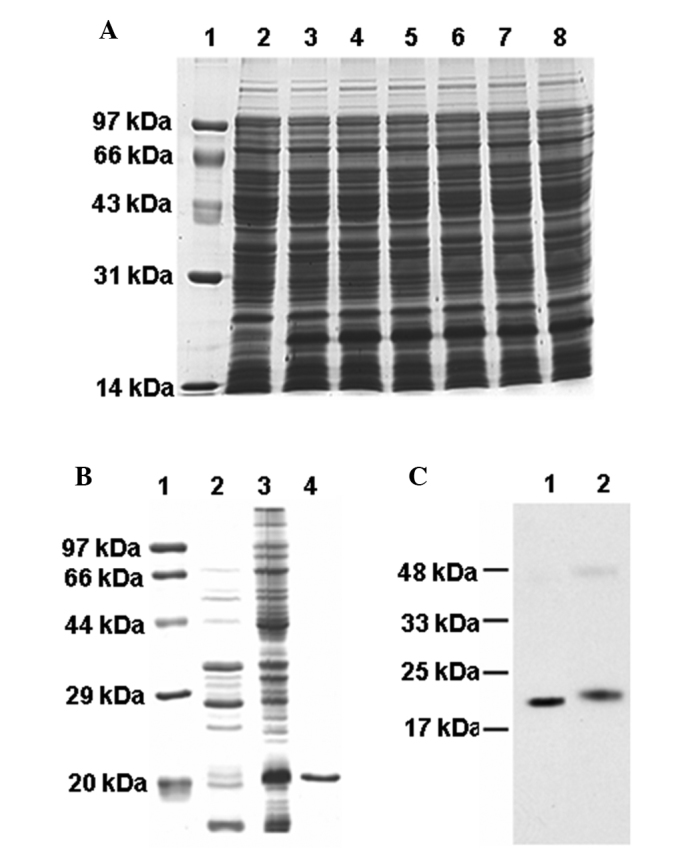
Expression and analysis of the mE7 protein. (A) Expression of the mE7 protein with IPTG induction. Lane 1, protein molecular weight markers; Lane 2, total protein extracts without IPTG induction; Lanes 3–8, total protein extracts with IPTG induction for 1–6 h, respectively. (B) Suspension and inclusion body of the mE7 protein following expression induction by IPTG for 4 h. Lane 1, protein molecular weight marker; Lane 2, suspension protein; Lane 3, inclusion body; Lane 4, purified mE7 protein. (C) Western blot analysis of (lane 1) purified mE7 and (lane 2) E7 protein. mE7, mutant non-transforming human papillomavirus 16 E7; IPTG, isopropyl-β-D-thiogalactoside.

The mE7 protein was expressed efficiently in *E. coli* Rosetta subsequent to 4-h induction with IPTG, which accounted for ~10% of total cell protein, as measured using densitometry (Fig. 2A). The mE7 protein was expressed as an inclusion body (Fig. 2B). The mE7 protein with the his-tag was purified using a Ni-NTA agarose column. The purification of the mE7 protein was confirmed by SDS-PAGE (Fig. 2B). The HPV16 mE7 and E7 proteins were each recognized by the human E7 antibody (Fig. 2C). The results of SDS-PAGE and western blot analysis revealed that the molecular weight (MW) of the purified mE7 was ~20 kDa, which was larger than the theoretical MW of 14.5 kDa, and the MW of the E7 protein was 20.5 kDa, which was also larger than the theoretical MW of 15 kDa (15). The mE7 protein was smaller than the E7 protein as it lacked of the five amino acids at the C-terminal end.

### Prevention of TC-1 growth by immunization with the mE7 protein

**Figure 3:**
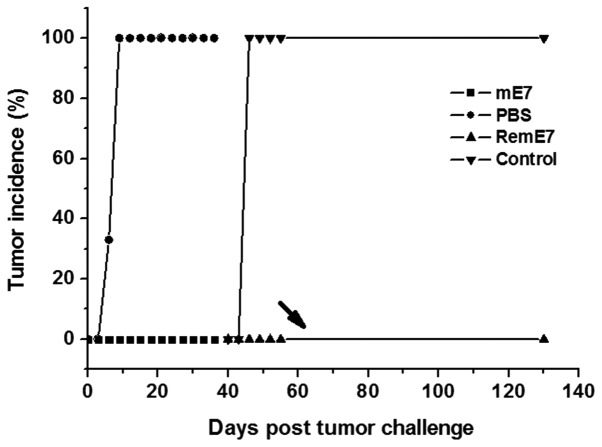
Prevention of TC-1 cell growth by immunization with the mE7 protein. The mice were immunized twice with non-transforming mE7 protein (■) or PBS (●), supplemented with either complete or incomplete Freund’s adjuvant, and the mice were then injected with 1×10^5^ TC-1 cells seven days later. After 40 days, tumor-free mice immunized with the mE7 protein and novel control group mice were re-challenged with 2×10^5^ TC-1 cells. These groups were termed the RemE7 (▲) and control groups (▼), respectively, with each group consisting of six mice. For 130 days, the tumor growth was monitored and the tumor incidence was also recorded. PBS, phosphate-buffered saline; mE7, mutant non-transforming human papillomavirus 16 E7.

Female C57BL/6 mice were immunized twice with either the mE7 protein or PBS. Subsequent to receiving an SC injection containing 1×10^5^ TC-1 cells, all control mice, which were immunized with PBS, developed tumors within nine days. However there no tumors were identified in mice immunized with the mE7 protein. After 40 days, the tumor-free mice and novel control group mice were re-challenged with 2×10^5^ TC-1 cells. All control mice developed tumors within six days, but no tumor was identified in the mE7 protein-immunized mice until 90 days subsequent to the administration of tumor cells (Fig. 3). These results demonstrated that immunization with the non-transforming mE7 protein was also able to elicit a long-term protective immunity against TC-1 tumor growth that was similar to the effect of immunization with the E7 protein (15).

### Inhibition of TC-1 cell growth by immunization with mE7

**Figure 4:**
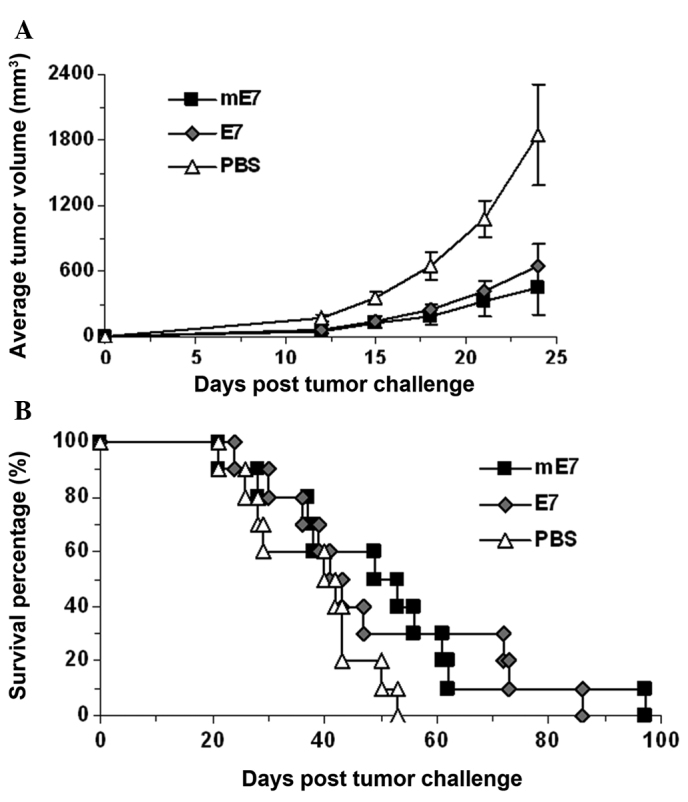
Inhibition of TC-1 growth by immunization with the mE7 protein. The mice were subcutaneously injected in the right flank with 1×10^5^ TC-1 cells, and then immunized with the mE7 protein, supplemented with complete Freund’s adjuvant, the following day. PBS and E7 were used as controls. Each group contained 10 mice. Seven days later, mice were immunized using the same solution, supplemented with incomplete Freund’s adjuvant. (A) Tumor growth was monitored every three days for 24 days and (B) the survival rate was recorded. Tumor growth was determined by measuring the maximal and minimal diameters with a vernier caliper, and tumor volumes were calculated as follows: volume = (length × width^2^) × 0.52. PBS, phosphate-buffered saline; mE7, mutant non-transforming human papillomavirus 16 E7.

Female C57BL/6 mice received an SC injection consisting of 1×10^5^ TC-1 cells in the right flank, and were immunized with the mE7 protein, E7 protein or PBS in combination with CFA the following day. Seven days later, the mice were immunized using the same solution supplemented with IFA. Each group contained 10 mice. The TC-1 cell growth was significantly inhibited in the mice immunized with mE7 or E7compared with the control group (P<0.05; Fig. 4A). However, there was no significant difference in the survival rate of the mice immunized with mE7 and the control group (P=0.055), or E7 and the control group (P=0.105; Fig. 4B). No tumor metastasis was observed in any mouse.

## Discussion

In the present study, immunization with the HPV16 mutant E7 protein was demonstrated to generate a long-term protective immunity against TC-1 tumor growth and significantly inhibited TC-1 tumor growth in the TC-1 mouse model.

Two HPV16 oncogenic proteins, E6 and E7, are co-expressed in the majority of HPV16-induced cervical cancer cells and therefore present good targets for therapeutic vaccines of cervical cancer (4,6,8). Our previous study reported that immunization with the full-length HPV16 E7 protein may elicit stronger immunological protection compared with the E6 protein, and results in a long-term specific protective immunity against TC-1 tumor growth (15). It has been reported that the transforming ability and trans-activation of the E7 protein was eliminated by the Cys-24 to Gly substitution (19). The disruption of the Cys-X-X-Cys motif that lies towards the carboxyl terminus of E7 protein appeared to result in a greatly impaired transforming ability and reduced transactivation (19). In the present study, the prokaryotic expression system for the mE7 protein, the pET-28a(+)-mE7 plasmid, was constructed in order to abolish the transforming ability of the E7 protein. Compared to the E7 protein, the mE7 protein was lacking residues 94–98 of the E7 protein, with Cys-94 being an amino acid residue in the carboxyl terminal Cys-X-X-Cys motif of the E7 protein, and residue 24 of the E7 protein was changed from Cys to Gly. Therefore, vaccination with the mE7 protein is safer compared with vaccination using the E7 protein.

The mE7 protein was further purified using a Ni-NTA agarose column, which was confirmed by SDS-PAGE and western blot analysis. The present results revealed that the MW of the mE7 protein was ~20 kDa, which is larger than the theoretical MW of 14.5 kDa, and the MW of the E7 protein was 20.5 kDa, also larger than the theoretical MW of 15 kDa (15). The cause of MW variation was the electrophoretic migration of the acidic E7 protein being altered by the positively-charged basal amino acids of the his-tag (24). The electrophoretic migration of the mE7 protein may possess the same property.

In order to determine the immunological effects of the HPV16 E6 or E7-associated vaccine, the TC-1 cell line was constructed by Dr Wu of Johns Hopkins University (21). In the present study, the TC-1 cervical cancer mice model was used to determine the effects of vaccination with the mE7 protein using CFA or IFA, and PBS was used as a control. To the best of our knowledge, the present results revealed for the first time that immunization with non-transforming mE7 protein may elicit the same protective and therapeutic immunity against TC-1 cell growth as the E7 protein in the TC-1 cervical cancer mice model. However, an adjuvant was required to facilitate the anti-tumor function of the mE7 protein. HSP has been reported to possess the potential to act as both antigen vector and adjuvant in enhancing antigen-specific tumor immunity (25,26). In a previous study, it was demonstrated that mouse autologous HSP70 enhanced the antitumor potency of the E7 protein without any adjuvant (16). The present results demonstrated that immunization with the HPV16 mE7 protein was able to elicit a long term protective immunity against TC-1 tumor growth and a significant inhibition of TC-1 tumor growth in the TC-1 mouse model. Therefore, the non-transforming mE7 and mouse autologous HSP70 recombinant proteins are hypothesized to also be able to elicit effective and safer anti-tumor activity compared with the HSP70-E7 recombinant proteins.

## Figures and Tables

**Table I:** Primer sequences of mE7 gene used for amplification.

Primer	Sequence	Restriction site
mE7p1	5′-CGCATATGGCTAGCATGACTGGTGGA-3′	*Nde*I
mE7p1-G-3′	5′-TAATTGCTCATAACCGTAGAGATCAGTTG-3′	None
mE7p2-G-5′	5′-CAACTGATCTCTACGGTTATGAGCAATT-3′	None
mE7p2	5′-CGAAGCTTTTAGATGGGGCACACAATTC-3′	*Hind*III

mE7, mutatant non-transforming human papillomavirus 16 E7.
